# An fMRI Study Exploring the Overlap and Differences between Neural Representations of Physical and Recalled Pain

**DOI:** 10.1371/journal.pone.0048711

**Published:** 2012-10-31

**Authors:** Merle Fairhurst, Katherine Fairhurst, Chantal Berna, Irene Tracey

**Affiliations:** 1 Nuffield Division Anaesthetics, Nuffield Department Clinical Neurosciences, Centre for Functional Magnetic Resonance Imaging of the Brain, University of Oxford, Oxford, England, United Kingdom; 2 Max Planck Institute for Human Cognition and Brain Sciences, Leipzig, Germany; National Research & Technology Council, Argentina

## Abstract

Implementing a recall paradigm without hypnosis, we use functional MRI (fMRI) to explore and compare nociceptive and centrally-driven pain experiences. We posit that a trace of a recent nociceptive event can be used to create sensory-re-experiencing of pain that can be qualified in terms of intensity and vividness. Fifteen healthy volunteers received three levels of thermal stimuli (warm, low pain and high pain) and subsequently were asked to recall and then rate this experience. Neuroimaging results reveal that recalling a previous sensory experience activates an extensive network of classical pain processing structures except the contralateral posterior insular cortex. Nociceptive-specific activation of this structure and the rated intensity difference between physical and recalled pain events allow us to investigate the link between the quality of the original nociceptive stimulus and the mental trace, as well as the differences between the accompanying neural responses. Additionally, by incorporating the behavioural ratings, we explored which brain regions were separately responsible for generating either an accurate or vivid recall of the physical experience. Together, these observations further our understanding of centrally-mediated pain experiences and pain memory as well as the potential relevance of these factors in the maintenance of chronic pain.

## Introduction

A growing body of neuroimaging literature has focused on and compared the neural correlates of simulated action and perception. These studies demonstrate that brain regions critically involved in motor, sensory and emotional processing can be activated without peripheral inputs [1]–[6]. Beyond this observed overlap, identification of distinct neural activation explains how imagined, internally generated events differ from a physical experience. Investigating pain in this manner is in its infancy, yet it provides a novel way of comparing peripherally and centrally mediated pain. As is thought to sometimes be the case for phantom-limb pain [7] or other chronic pain states [8], central mechanisms are able to act independently of peripheral nociceptive input to create a vivid pain experience. It is therefore of particular clinical relevance that we explore the process by which internally generated experiences of pain are created.

Some groups have employed hypnosis to induce imagined or suggested sensations of pain in the absence of nociceptive input, and results suggest some overlap with neural activity during physical pain [9]–[12]. Krämer and colleagues explored the effect of prior allodynic experience on brain responses to tactile-stimulation and similarly showed that some brain regions involved in pain processing were activated when subjects attempted to imagine the stimulus as allodynic [12]. In the related field of empathy, both perspective switching [13] and embodied empathising [14] have been used to explore the perception of pain and the subsequent neural activation of pain processing regions in the absence of a nociceptive input.

As an alternative approach, our paradigm uses a pain recall task, a form of mental imagery, that avoids the need for hypnosis or confounds of empathy-related processing. Mental imagery techniques have previously been shown to be useful in eliciting analgesic responses, with these methods focusing on the powerful effect of suggestion [15]. Based on phenomenological evidence and numerous behavioural studies, we know that vivid memories of pain events can be readily retrieved [16]–[18]. However, due to the complex nature of both pain and memory processing, there are relatively few neurophysiological studies that have ventured to explore these intricately related processes concurrently [19]. In the present study, we use a parametric design and focus on the link between varying the intensity of physical pain events and subsequent recall. In so doing, we test the hypothesis that the quality of the pain memory and the related brain activity will depend on the intensity of the noxious pain event that is recalled. More generally, it has been suggested that a more painful stimulus, perhaps due to a higher threat or emotional value, would be encoded more strongly than a mildly painful or non-painful, unthreatening event [16], [20], [21]. We therefore have acquired subjective ratings of recalled intensity and vividness, and expect that depending on the level of thermal input, recall will be rated as more or less intense and the experience will be more or less vivid.

**Figure 1:**
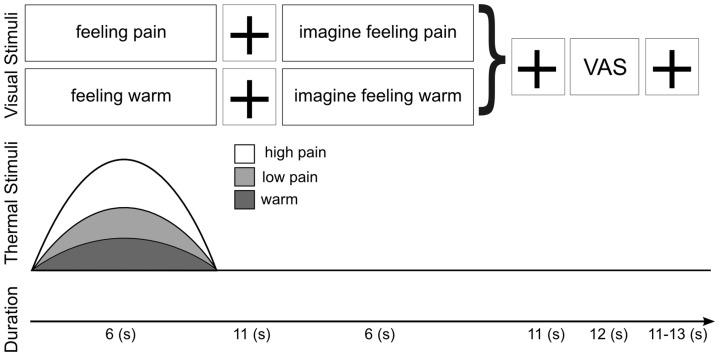
Study design for creating a recalled pain experience based on a preceding nociceptive event. Each block consisted of a six-second, thermal stimulus, either warm, low pain or high pain. The thermal stimulus was produced by an in-house built thermode which could ramp up to the targeted temperature in 1 second (bell shaped-stimulus for illustration purpose only).For the full duration of the thermal stimulus, a verbal visual stimulus “feeling pain” was shown. This was followed by a delay (time-to-test interval) of eleven seconds during which a fixation cross was presented. Subjects were then instructed by means of a visual stimulus “imagine feeling pain” to recall and imagine the preceding thermal event. This recall was performed in the absence of peripheral somatosensory stimulation. After each recalled pain event, subjects used a visual analogue scale (VAS) to rate the intensity and vividness of the “imagined pain” event. (For further details, refer to Materials and Methods.).

Beyond this, we wanted to determine whether one could simulate pain as a near sensory experience if the recalled event occurred temporally close to the original painful stimulus. Several studies have suggested that a memory “trace” of a painful experience may exist, but in a limited form such that sensory re-experiencing is not available after a painful event [16], [22], [23]. This has been further explained with suggestions that sensory information, such as accurate pain intensity levels, is not retained in long-term memory but exist as part of a short-term mental trace that degrades over time [16], [17], [19], [22]. We therefore posit that, if the time-to-test interval is sufficiently short, it is likely that an intact memory trace exists from which the painful experience is recreated [17], [19], [24]. Therefore using functional MRI (fMRI), we used nociceptive and imagined recalled pain events to identify and contrast brain structures specific to either peripherally initiated or centrally generated pain experiences. We further explored which brain regions were separately responsible for generating either an intensity-accurate or vivid recollection and simulation of pain.

**Table 1:** Neural activation during recalled pain.

*Recalled Low Pain > Baseline*							
	Z score		Coordinates				
			x		y		z
anterior insula (left)	4.81		36		10		−4
anterior insula (right)	4.59		−38		12		−4
basal ganglia (left)	4.21		−26		10		4
basal ganglia (right)	4.1		16		−2		4
thalamus	4.05		8		−14		4
midcingulate	4.59		−2		12		28
SI	3.86		44		−14		40
MI (right)	2.37		48		−6		36
inferior parietal lobule (right)	2.6		54		−42		28
visual cortex	3.78		10		−94		−12
cerebellar crus II (left)	4.14		−20		−72		−36
*Recalled High Pain > Baseline*							
dorsolateral prefrontal cortex	4.24	−34		48		16	
midcingulate	5.75	4		10		40	
anterior insula L	4.02	−30		18		4	
anterior insula R	5.74	34		10		4	
basal ganglia (left)	4.82	24		0		2	
basal ganglia (right)	4.61	−26		0		2	
thalamus	4.12	12		−16		4	
midcingulate	4.60	0		20		30	
SI	3.91	40		−14		36	
SII (right)	3.74	50		−26		18	
MI/premotor cortex	5.89	−34		−4		52	
SMA	5.63	4		−4		56	
inferior parietal lobule (left)	3.22	56		−36		40	
inferior parietal lobule (right)	3.21	−38		−58		46	
precuneus	3.74	−8		−72		46	
cerebellar crus I/VI (left)	3.41	−32		−58		−32	
cerebellar crus I (right)	4.00	28		−64		−32	
cerebellar crus II (right)	3.54	8		−76		−32	

Coordinates of structures and associated peak voxel Z-scores more active during recalled pain events relative to baseline. Coordinates in MNI space. p<0.01 corrected for multiple comparisons.

**Table 2:** Comparing recalled pain (high pain) and recalled warm conditions.

*Recalled High Pain – Recalled Warm*				
	Z score	Coordinates		
		x	y	z
thalamus (right)	3.41	6	−20	0
thalamus (left)	3.28	−5	−20	2
anterior cerebellum	3.31	0	−54	−20
posterior cerebellum	3.21	0	−64	−24
deep cerebellar nuclei (midline)	2.56	0	−54	−26
SI (left)	2.73	−10	−30	54
premotor cortex (left)	2.83	−4	6	48

Coordinates of structures significantly more active during “imagine feeling pain” events (“imagine feeling pain” (high pain) > “imagine feeling warm” (high pain). Coordinates in MNI space and associated peak voxel Z-scores. p<0.01 corrected for multiple comparisons.

## Materials and Methods

### Subjects

15 healthy volunteers (nine females and six males; age range: 21–42; mean age: 28, SD = ±5.9) were recruited after screening for absence of any prior history of pain, neurological or psychiatric disorders, and not meeting any of the exclusion criteria for MR experimentation. Written informed consent was obtained in accordance with the Declaration of Helsinki and the study was approved in full by the Central Oxfordshire Research Ethics Committee (C02.086).

**Figure 2:**
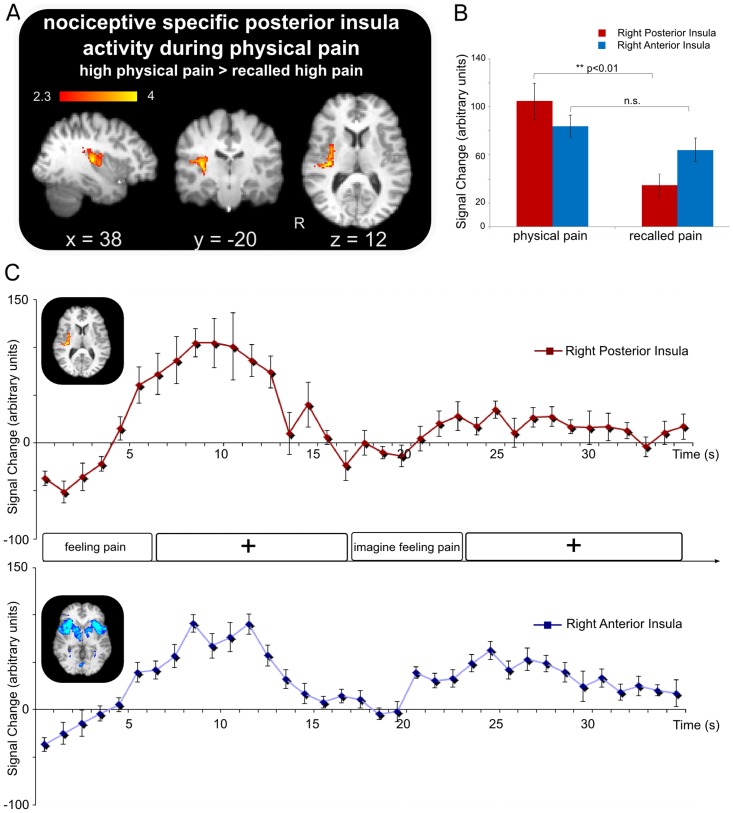
Nociceptive specific activity in the posterior insula. A) Neural activity specific to processing physical pain (high physical pain > recalled high pain). Group contrast, mixed effects, Z = 2.3; p = 0.01, see Table 3 for further details of coordinates. B) Group mean parameter estimates of peak activity during physical and recalled pain describing observed nociceptive specific activation of posterior insula as compared to anterior insula. Error bars represent standard error. C) Timecourse analysis of posterior and anterior insula showing mean signal change across “Feeling pain” and “Imagine feeling pain” events. Insets shows results of (top) posterior insula activity from a contrast of high physical pain > recalled high pain (see Table 3) and (bottom) anterior insula activity as revealed by a conjunction analysis of common activity across the physical and recalled high pain conditions (see Table 5 for further details of coordinates). Error bars represent standard error.

**Table 3:** Group contrast (mixed effects) comparing high physical pain and recalled high pain conditions.

*High Physical Pain > Recalled High Pain*				
	Z score	Coordinates		
		x	y	z
posterior insula (right)	3.81	38	−20	12

Coordinates of structures significantly more active during “feeling pain” (high pain) events (“feeling pain” (high pain) > “imagine feeling pain” (high pain). Coordinates in MNI space and associated peak voxel Z-scores. p<0.01 corrected for multiple comparisons.

**Table 4:** Neural activity specific to perceiving a physical painful stimulus.

*Physical (high pain-warm) > Recalled (high pain-warm)*				
	Z score	Coordinates		
		x	y	z
thalamus (right)	3.24	14	−18	−6
thalamus (left)	3.00	−12	16	6
PAG (right)	2.82	6	−32	−6
midcingulate	3.06	14	−18	6
posterior insula (right)	3.76	34	−2	12
posterior insula (left)	2.99	−38	−2	8
anterior insula (right)	2.75	34	16	4
anterior insula (left)	2.9	−32	6	8
basal ganglia – putamen (right)	2.59	26	2	6
SII (right)	2.86	46	−30	20

Coordinates of structures whose activation is significantly greater in physical high pain (relative to control physical warm condition) versus recalled high pain (relative to control recalled warm condition). Coordinates in MNI space and associated peak voxel Z-scores. p<0.01 corrected for multiple comparisons.

**Table 5:** Group contrast (mixed effects) conjunction analysis.

*Conjunction: High Physical Pain and Recalled High Pain*				
	Z score	Coordinates		
		x	y	z
thalamus (right)	5.39	12	−22	6
thalamus (left)	5.07	−10	−18	6
anterior insula (right)	5.59	34	8	8
anterior insula (left)	5.48	−34	10	4
Midcingulate	5.58	0	10	32
parietal cortex (right)	5.02	24	−54	36
parietal cortex (left)	5.09	−38	−50	36
SI (left)	4.76	−26	−36	54
dlPFC (left)	5.19	−42	40	6
cerebellum Crus I (right)	5.46	−38	−68	−38
cerebellum Crus I (left)	5.19	36	−70	−38
premotor cortex (right)	5.93	12	−2	64
premotor cortex (left)	4.54	−16	−4	64
visual cortex (right)	3.88	16	−80	0
visual cortex (left)	3.47	−18	−80	0

Coordinates of structures commonly activated during “feeling pain” and “imagine feeling pain” (high pain) events. Coordinates in MNI space and associated peak voxel Z-scores. p<0.01 corrected for multiple comparisons.

### Study Design

The imaging session consisted of two blocks of 15, six-second thermal stimuli to the dorsum of their left hand. Subjects were presented with three types of thermal stimuli (warm, low pain and high pain) that had previously been defined by subjective thresholding in the scanner. Warm was defined as 0/10, low pain was defined as 2/10 and high pain was defined as 6–7/10 (on a verbal numerical scale from 0–10, where 0 is not painful, 1 is the pain threshold and 10 is extremely painful). Overall, there were 10 repeats for each stimulus type, pseudo-randomized over the two imaging blocks. Each repeat consisted of a visual stimulus reading either “Feeling warm” or “Feeling pain,” during which the individual would receive either a warm or noxious thermal stimulus. An eleven second gap (accompanied by a fixation cross) followed. The length of this “time-to-test” interval was chosen based upon data suggesting it would allow accurate pain memory recall [17] and that it would be sufficient to allow the haemodynamics of blood flow associated with brain activity to normalise [25]. Subjects were then asked to recall and imagine the preceding stimulus while viewing a visual stimulus that read either “Imagine feeling warm” or “Imagine feeling pain” (Figure 1).

**Figure 3:**
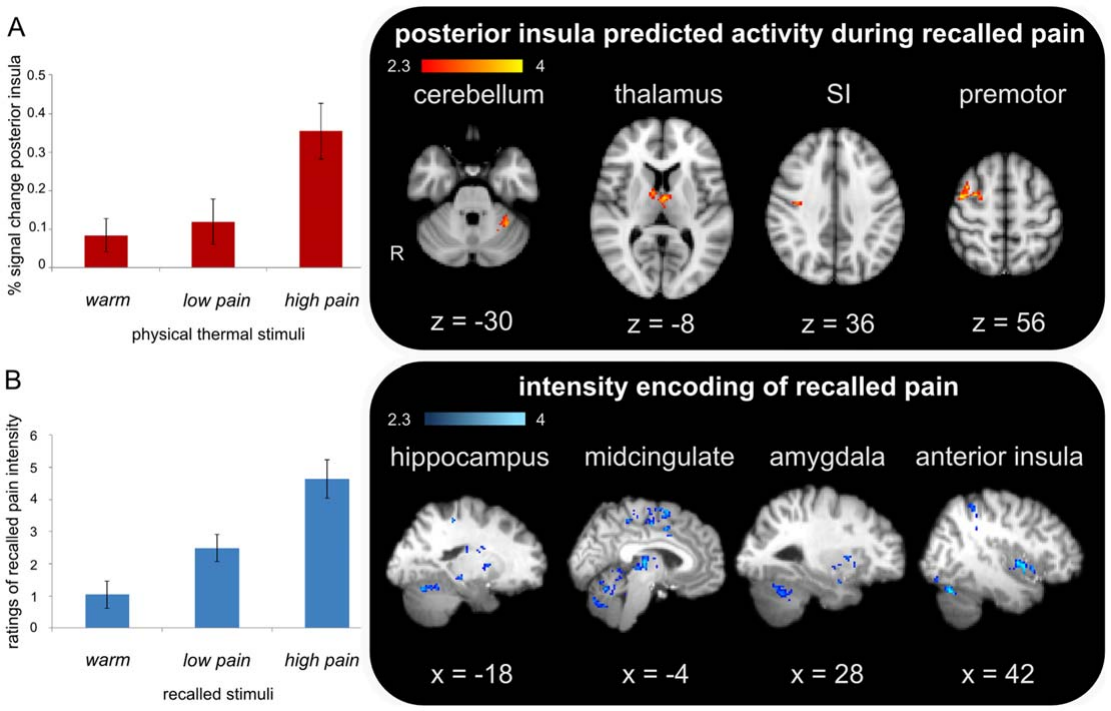
Intensity and recalled pain. A) Linking nociceptive intensity and therefore posterior insula activity during physical pain with neural activation during subsequent recall. Left: graphical summary of group mean parameter estimates (PE) of percent signal change in posterior insula during high physical pain. Error bars represent standard error. Right: predicted activity during recalled high pain incorporating individual PEs as a regressor. See Table 6 for further details of MNI coordinates. B) Intensity coding during recalled pain. Left: graphical summary of recalled intensity ratings across conditions of imagined stimuli. Error bars represent standard error. Right: Brain regions from a whole brain search whose activity increases with increased perceived intensity of the imagined stimulus and are not seen in the similar analysis performed to explore vividness encoding. Group contrast, mixed effects, Z = 2.3; p = 0.01. See Table 7a for full list of activation with MNI coordinates.

**Table 6:** Activation during recalled pain predicted by posterior insula activation during physical pain.

*Recalled pain as predicted by increasing nociceptive input during physical pain*				
	Z score	Coordinates		
		x	y	z
thalamus (right)	3.17	8	−6	10
thalamus (left)	3.98	−2	−10	10
anterior cerebellum (right)	3.21	18	−38	−24
anterior cerebellum (left)	3.86	−24	−52	−24
PAG (right)	3.16	4	−34	−6
SI (right)	3.35	44	−12	42
premotor cortex (right)	3.42	42	−4	48
*Recalled pain as predicted by decreasing nociceptive input during physical pain*				
dorsolateral prefrontal cortex	3.32	−26	48	36
cingulate	4.74	−10	44	20

Coordinates of structures whose activation is significantly correlated with increasing PE s of posterior insula activity during physical pain events. Coordinates in MNI space and associated peak voxel Z-scores. p<0.01 corrected for multiple comparisons.

**Table 7:** Function fitting analysis.

*Function Fitting Analysis*				
a) Intensity		Coordinates		
	Z score	x	y	z
mid-cingulate	2.53	−4	8	36
premotor cortex (right)	3.1	2	−12	62
parietal cortex (right)	3.39	38	−50	54
thalamus (right)	3.5	6	−20	4
thalamus (left)	3.58	−6	−20	4
PAG (right)	2.06	4	−32	−4
Hippocampus (left)	2.39	−18	−26	−12
putamen (right)	2.34	28	8	−2
putamen (left)	2.34	−32	0	−2
anterior insula (right)	3.34	42	2	−2
anterior insula (left)	2.29	−40	10	−2
SII (right)	3.2	58	−30	22
amygdala (right)	2.04	26	−2	−20
cerebellum midline deep nuclei	2.31	0	−52	−26
cerebelum lobule VI	3.36	18	−60	−26
cerebellum crus I (right)	3.05	40	−66	−32
cerebellum crus I (left)	2.36	−24	−76	−32
**b) Vividness**				
premotor cortex (left)	2.7	−2	0	52
thalamus (right)	3.46	6	−20	4
thalamus (left)	3.22	−10	−22	4
cerebellum crus I (right)	2.73	40	−62	−30
cerebellum crus I (left)	2.66	−26	−74	−30
cerebellum lobule VI	3.06	26	−54	−30
cerebellum deep nuclei midline	2.19	2	−52	−26
putamen (right)	2.2	32	−8	−4
caudate (right)	2.85	18	0	16

Group contrast (mixed effects) of intensity and vividness encoding of imagined events. Results for a search of voxels whose behaviour correlated with the observed trend in psychophysical data of group mean imagined stimulus ratings of a) intensity and b) vividness. Coordinates in MNI space and associated peak voxel Z-scores. p<0.01 corrected for multiple comparisons.

During the recalled event, subjects were instructed to visualise the location of the previous thermal stimulus, changes in intensity, and the context surrounding the thermal experience while attempting to simulate the sensation. Using a visual analogue scale (VAS), subjects were then instructed to use a slider to rate both the *intensity* of the recalled event and the *vividness* of the evoked sensation, measuring how close to reality it felt. At three points during the study, subjects were asked to rate the stimulus intensity of the real thermal event using a VAS. The three thermal conditions were pseudo-randomised both within the experiment and across subjects.

### Stimuli

#### Thermal noxious stimuli

A thermal resistor developed in-house and controlled by in-house written software was used to increase skin temperature. The device's ramp rate is such that the desired stimulating temperature is reached within one second of the triggered event and sustained evenly for the remaining five seconds of the stimulus. The device records the varying temperature at the site of stimulation demonstrating a return to baseline within six seconds. This device has been reliably used within several previous studies [26], [27]. Before the experiment, all subjects were thresholded in a stepwise fashion (0.5°C–1°C) to find individual ratings of 0/10 (warm), 2/10 (low pain), and 6–7/10 (high pain). A one-minute interval was allowed between each six-second stimulus to ensure the safety of the skin, during which the subjects gave a verbal rating for the previous stimulus. Temperatures applied across subjects were warm, non-noxious condition: mean ± SD  = 43.45°C±1.0, “low pain” condition: mean ± SD  = 47.44°C±2.1 and “high pain” condition: mean ± SD  = 49.86°C±2.

#### Visual stimuli

Visual stimuli included a fixation cross and four six-second prompts: “Feeling warm,” “Feeling pain,” “Imagine feeling warm” and “Imagine feeling pain”. Visual analogue scales (VAS) were presented to obtain online ratings for intensity of recalled pain and vividness. Each scale was presented for six seconds. The “Imagined intensity” scale was anchored with no pain at the minimum and extremely painful at the maximum. Similarly, vividness was anchored with not vivid and extremely vivid. All visual stimuli were projected onto a screen visible to the subject via prism glasses. Visual stimulation was continuous throughout the experiment.

### MRI Data acquisition

Functional imaging was conducted using a 3 Tesla Siemens/Varian MRI system with a bird-cage radio frequency coil and four channel phased-array receiver coil. A gradient echo-planar imaging (EPI) sequence was used with a TR  = 3 s; matrix  = 64×64; TE  = 30 ms; 41×3 mm axial oblique slices; volumes  = 294; FOV = 192×192; voxel size  = 3×3×3 mm^3^. Scans were acquired continuously throughout the experiment. High resolution, T1-weighted, structural scans (64 slices at 1×1×1 mm^3^ voxel size) were obtained for each individual for anatomical overlay of brain activation.

### Data Analysis

#### Psychophysical Data

Online ratings for intensity of recalled pain and vividness of recalled pain were grouped according to the preceding physical stimulus given (warm, low pain and high pain) and the individual means and standard deviations calculated. One-way ANOVAs were used to test for statistical difference between the average ratings for all three conditions, for both vividness and intensity across individuals. Individual ratings across conditions of vividness and intensity ratings were correlated using Pearson correlation coefficient. Temperatures of the thermode at the skin surface were recorded once every 500 ms. The mean and standard deviation of these temperatures across epochs prior to and during the recalled events were calculated per individual and then across subjects.

#### Imaging Data

Analysis of all neuroimaging data sets was performed using FEAT (FMRIB Expert Analysis Tool) Version 5.63, part of FSL (FMRIB's Software Library, www.fmrib.ox.ac.uk/fsl). Pre-statistic processing included: motion correction using MCFLIRT (Motion Correction FMRIB's Linear Image Registration tool, [28]), non-brain removal using BET [29], spatial smoothing using a Gaussian Kernel of 4 mm full width at half-maximum and non-linear high pass temporal filtering (Gaussian-weighted least-squares straight line fitting, with sigma = 40.0 s). Registration included co-registration of the functional scan onto the individual T1 high-resolution structural image and then registration onto a standard brain (Montreal Neurological Institute MNI 152 brain) using FLIRT (FMRIB's Linear Image Registration Tool, [28]). Statistical analysis at the first, individual subject level was carried out using a general linear modelling (GLM) approach [30]. Time-series statistical analysis was carried out using FILM (FMRIB's Improved Linear Model) with local autocorrelation correction [25]. Second level analysis grouped the data of each subject's two scanning blocks, using the data from the first level of analysis. For group statistics, analysis was carried out using FEAT (FMRI Expert Analysis Tool) with higher-level analysis carried out using FLAME (FMRIB's Local Analysis of Mixed Effects). This analysis method allows for incorporation of variance within session and across time (fixed effects) and cross session variances (random effects). Cluster thresholding was performed with a Z-threshold of 2.3 and a corrected p-value of <0.01 with a cluster-based correction for multiple comparisons using Gaussian Random Field Theory [30], [31]. Contrasts performed explored activation during the three types of physical and recalled events compared to baseline, as well as a 1×3 repeated measures ANOVA (of imagined events) and paired t-test subtractions between real and recalled conditions. Further analyses included region of interest (ROI) analysis of posterior and anterior insula cortices to explore their relative timecourses as well as to extract individual parameter estimates (PEs). These PEs were divided by condition of thermal input (warm, low pain, high pain) and used as a regressor predictive of neural activation during recalled events (whole brain search). Paired t-tests of peak-to-peak (physical high pain versus recalled high pain) activity was performed across individual PEs in these two structures describing the nociceptive specific response of the posterior insula. A conjunction analysis was performed with contrasts ‘physical pain vs. baseline’ and ‘imagined pain vs. baseline’ to define regions active during both periods [32]. This analysis, based on inclusive masking, constitutes a true logical ‘AND’ operation. To explore activity specific to either intensity recall or vividness, a function fitting analysis was performed. Second level contrasts of parameter estimates (COPEs) for the three recalled conditions (warm, low and high pain) were inputted for all subjects into a higher-level analysis. An additional explanatory variable (EV) was created weighting the conditions according to the function of subjective intensity and vividness ratings of the recalled events. Weightings for intensity were −1, 0, +1 representing the linear function observed across the imagined conditions. In a similar and separate analysis, weightings for the function of the vividness ratings were incorporated describing the observed trend of equal vividness for warm and low pain and higher vividness for high pain (−1, −1, +2). This analysis therefore identifies voxels that significantly follow the same pattern of behaviour.

## Results

### Psychophysical data

In post-scan debriefing sessions, all of the subjects reported the ability to recall the thermal events during the cued “imagined” conditions. The ability of healthy volunteers to recreate a similarly intense experience was demonstrated by the fact that ratings for intensity of recalled pain were related to the actual stimulus applied (significant increase in imagined intensity rating with graded thermal events). Ratings for intensity of recalled sensory events showed a significant positive linear trend across intensities one-way ANOVA: F_(2,42)_ = 10.98, p<0.01; Intensity: recalled warm mean ± SE  = 1.08±0.43, recalled low pain mean ± SE  = 2.52±0.42; recalled high pain mean ± SE  = 4.49±0.6).

Furthermore, the significant difference between imagined low and imagined high pain ratings, despite the lack of a cue for this difference, confirms that subjects accurately recreated and matched the recalled event to the prior sensory experience. Despite all three conditions being rated as significantly different for intensity, vividness ratings reveal that subjects were equally able to imagine warm and low pain stimuli, whereas “high pain” stimuli were rated as significantly more vivid (one-way ANOVA: F_(2,42)_ = 2.32, p = 0.11 Vividness: warm mean ± SE  = 4.33±0.53, low pain mean ± SE  = 4.27±0.47; high pain mean ± SE  = 5.66±0.46).

To explore the perceptual differences between physical and recalled pain events, subjective ratings of the thermal stimuli were acquired at three time points during the study (Real pain intensity: warm mean  = 0; low pain mean ± SD  = 2.2±0.47; high pain mean ± SD  = 6.29±0.6). A comparison with rated intensity of imagined events revealed a significant difference between physical warm vs. recalled warm (t_(14)_ = −2.364, p<0.05) and physical high pain vs. recalled high pain (t_(14)_ = 2.844, p<0.05). No significant difference was observed between physical low pain vs. imagined low pain (t_(14)_ = −0.603, p = 0.556). Individual averages were taken of the recorded temperatures of the stimulating thermode prior to and during the recalled events to ensure that nociceptive fibres were not stimulated (mean ± SD temperature at onset across subjects and across imagined conditions  = 37.8±1.2°C).

### Imaging Data

#### Neural activation during recalled pain events

Beyond activation of pain processing structures, in both imagined pain conditions (recalled low pain > baseline contrast and recalled high pain > baseline contrast – Table 1), activity was observed in areas including the anterior insula, basal ganglia, parietal and prefrontal cortices. From our behavioural data we posited that recalling a painful event, rated as significantly more intense and more vivid, should differ neurally from recalling a warm, non-noxious stimulus. A subtraction analysis of recalled high pain from recalled warm was therefore performed revealing more focused activity in areas commonly associated with mental imagery as well as pain processing including the SI, premotor cortex, thalamus, PAG and the cerebellum (Table 2).

#### Neural activation specific to noxious thermal events

Based on acquired intensity ratings, it was assumed that feeling (physical) pain should differ from recalling pain. This difference was investigated using a mixed effects analysis group contrast of physical high pain versus imagined high pain which revealed nociceptive-specific activity in only one brain region: contralateral (right) posterior insula (Figure 2A, Table 3). This result suggests that the neural response related to the original physical event is highly similar to the recalled trace with overlapping activation in all areas but one, possibly accounted for by the difference in perceived intensity between the physical and recalled events. When we control for the effect of non-painful sensory processing (subtraction of physical high pain versus warm > imagined high pain versus warm), we observe a greater mismatch in responses to pain following nociceptive and recalled stimuli (Table 4). The data suggest that processing of the pain related information during the physical event involves quantitatively greater activity in areas including SII, cingulate, thalamus and PAG and possibly unique activity in the posterior insula. A reverse contrast (subtraction of recalled high pain versus warm > physical high pain versus warm) reveals no activation specific to recalled imagined pain.

#### Differences, overlap and links between physical and recalled pain

Consistent with our hypothesis that the recalled task draws upon a trace of the original nociceptive event, a conjunction analysis of recalled and physical pain conditions revealed significant activation in most pain processing structures including bilateral anterior insula, ACC, thalamus and SI (Table 5), suggesting more extensive overlap than described in previous studies. As discussed above, however, our data highlight a difference between a nociceptive specific response in the posterior insula and a more general pain response as elicited by both physical and imagined pain events. To illustrate this point, a comparison of the activation profiles of the anterior and posterior insular cortices revealed that while the posterior insula is significantly more active during physical pain (mean ± SE: physical pain = 104.66±14.96, recalled pain = 34.84±9.30; paired t_(14)_ = 3.0527, p<0.01), the anterior insula shows two separable but comparable BOLD responses to the physical and recalled pain events (mean ± SE: physical pain = 83.89±9.26, recalled pain = 64.07±9,75; n.s. paired t_(14)_ = 1.7, p>0.05 see Figure 2B). Timecourse analyses serve to demonstrate that activation during imagined pain is not merely due to a spillover-effect following the nociceptive event but rather that the peak seen in anterior insula during recall arises following a recovery period (Figure 2C).

Our trace hypothesis however, suggests a link between these two events. As such, we posited that a greater response during the original stimulus should predict greater activation during recall. By extracting PEs from the posterior insula (during physical high pain – Figure 3A left), we explored correlated activity during subsequent recall. This analysis revealed that with increasing posterior insula activity during physical pain, increased activation of SI, premotor cortex, PAG, thalamus and cerebellum during recalled imagined events is observed (Figure 3A-right, Table 6). A reverse contrast correlating with lower levels of posterior insula activation during physical pain shows the recruitment of dorsolateral prefrontal cortex and midcingulate (Table 6).

We posit that both the extent of the overlap and the link between physical and recalled events, facilitated by the short time to test interval, underlies the reported intensity of sensory re-experiencing. To explore the neural signature of this element of recalled pain processing, we correlated both intensity and vividness ratings with our imaging data using a function fitting analysis. In this analysis, the whole brain volume was searched for voxels, which across the group of subjects, behaved according to the pattern observed across recalled conditions as dictated by the behavioral data. In the case of *intensity*, the function the analysis searched for was a linear increase across conditions (Figure 3B-left). Results from this analysis revealed activation in structures including bilateral thalamus, mid-cingulate, cerebellum, PAG, putamen, primary motor cortex, bilateral anterior insula, SII, hippocampus, amygdala, and right PPC (Figure 3B-right, Table 7a). A similar analysis was applied using a function that fit the pattern of activation following the trend observed in the *vividness* ratings. Despite a similar activation pattern correlating with vividness ratings across conditions (Table 7b), activity in previously identified memory structures including the anterior insula, midcingulate, hippocampus and the amygdala was specific to intensity encoding.

## Discussion

This study demonstrated that healthy volunteers, in the absence of hypnosis, could reproducibly recall and create internally-generated experiences of pain. The data suggest that our chosen time-to-test interval allowed subjects to consistently perceive and rate the recalled imagined experience in terms of certain sensory qualities. Specifically, subjective reports of recalled pain intensity were acquired and showed a parametric relation to the intensities of the preceding thermal stimuli, with all subjects reporting a significant difference between low and high pain conditions despite the lack of a cue for this difference. This is evidence for a strong dependence of the recalled event on the quality of the original stimulus. In line with previous studies, our imaging data reveal significant overlap between physical and recalled events suggestive of the use of a mental trace to recreate the internally generated experience of pain during recall. Our findings highlight several interesting distinctions between neural processing of nociception and centrally mediated pain. We show that recalled imagined pain activates core pain areas but that these are predominantly more active during physical events, presumably as a result of physical pain being subjectively perceived as more intense. Furthermore, we identify in a contrast of physical and recalled pain events that the activation profile of the contralateral posterior insula is nociceptive specific. To investigate the link between the recalled event and the intensity of the original stimulus, we extracted measures of posterior insula activity to explore the predicted activation during recalled imagined pain. In so doing, we identify a core set of areas that we believe allow for the subjectively rated sensory re-experiencing perceived by our subjects. We describe the qualities of this recalled imagined experience in terms of perceived intensity and vividness. Our data show how these two components represent intrinsically different aspects of the recalled imagined experience and identify unique activation of memory structures specific to processing of intensity-related recall.

### Recalled imagined pain activates an extensive brain pain relevant network except posterior insula

A group contrast (mixed effects) exploring the difference between physical and imagined pain (high physical pain > imagined high pain) revealed isolated activity in only one brain region: contralateral posterior insula (Figure 2A, Table 3). The posterior insula has been noted as an area most frequently activated in pain experiments [33], however the precise role of this structure in pain processing is still under discussion. Work by Frot and colleagues explored differential intensity encoding between posterior insular cortex and second somatosensory cortex (SII), suggesting that the posterior insula demonstrated a unique pain level related response, with no saturation effect, perhaps allowing for subsequent triggering of affective recognition and/or a motor reaction to noxious stimuli [34]. In the present study we show that extracted mean PEs for the posterior insula show a graded response to physical stimulus intensity (Figure 3A-left). This supports the notion that the posterior insula, as suggested by Craig [35], is devoted to pain and temperature sensitivity, as it receives input from and is connected to a variety of pain related areas, such as parietal cortices, striatum and lateral prefrontal areas [36]. In support of this theory, direct electrical stimulation of the upper posterior part of the insular cortex results in a lateralised perception of pain contralateral to the side of stimulation [37]. Equally, Greenspan and colleagues reported that lesions in the posterior insula elevated pain thresholds contralateral to the side of the lesion [38]. Together these results fit with our findings that the posterior insula plays a critical role in peripheral nociceptive processing.

### Cortical and subcortical pain-related brain activity is not nociceptive-specific

Supporting the mixed effects group contrast data (physical pain > imagined pain), when commonalities between recalled imagined and physical pain conditions were further explored using a conjunction analysis, it revealed activation of several of the classical pain processing structures (Table 5), with more extensive overlap than has previously been documented [10], [11]. This confirms the hypothesis that a recalled imagined pain event, in the absence of peripheral nociceptive input, is sufficient to elicit extensive brain activation encompassing the entire established pain network except the posterior insula.

### Trace-based simulation of pain

A contrast identifying neural activation specific to recalling a painful event (imagined high pain > imagined warm) revealed activity in the thalamus, premotor cortex, SI, PAG, and the cerebellum (Table 2). This overlap with pain processing structures supports our theory that recalled pain is based upon or represents a trace of the preceding noxious event. Additionally, we show that the level of nociceptive input as indicated by posterior insula activation is predictive of correlated activation during recalled pain of similar areas including the SI, premotor cortex, thalamus and cerebellum (Figure 3A, Table 6). A related study reported SI activity during a memory delay period at an enhanced level compared with control trials [19] corroborating with accounts that SI is a site of transient storage of a tactile memory trace [39]. In terms of the somatosensory component of perceived pain, the present data reveal an interesting difference between physical and recalled imagined pain with SI (predominantly ipsilateral to stimulated hand) being observed in recalled imagined pain (relative to imagined warm) and predicted activity during recalled imagined pain by posterior insula activation, while SII (contralateral to stimulated hand) was seen as significantly more active in physical pain (physical: high pain-warm > imagined: high pain-warm).

Beyond activation of pain processing structures, in both imagined pain conditions (imagined low pain > baseline contrast and imagined high pain > baseline contrast – Table 1), activity was observed in many areas involved in working memory including the basal ganglia [40]–[42], parietal and prefrontal cortices [19], [43], [44]. In particular, dlPFC activity may be related to working memory tasks that require a high degree of executive control and more specifically in prioritising information for effective pain modulation [43]. It is interesting to note that a study of grey matter variation reported a significant difference in dlPFC density in chronic pain patients [45]. Furthermore, consistent with theories of a parietal and pre-frontal working memory network, prefrontal and right posterior parietal cortex were active in all of the recalled conditions [46]. Within the theoretical framework of a trace-driven recalled imagined event, the importance of experience-based simulation may explain the observed activity of a working memory network. Interestingly, the dlPFC and cingulate were identified in a reverse contrast of correlated activation during recalled imagined pain predicted by lower levels of posterior insula activity during physical pain (Table 6). This is possibly suggestive of greater effort required to create the imagined pain event.

### Intensity and vividness of imagined events

Confirming a report by Morley [16], our data support the idea that intensity and vividness are related but represent intrinsically different aspects of recalled imagined pain. Our function-fitting analysis exploring the neural activation specific to intensity (accuracy of recall) and vividness of the recalled imagined experience reveal that despite a large amount of overlap, activity in the midcingulate, anterior insula, amygdala and hippocampus was unique to intensity encoding (Figure 3B, Table 7a). These structures are characteristic of memory encoding and therefore may be indicative of memory-related processing essential for recalling and recreating an imagined experience of matched intensity [47]. Activation of the anterior insula was observed in all recalled conditions, consistent with recent studies on pain recall [44] where a role in relaying information from somatosensory cortices for short-term retention in the frontal cortex was suggested [19]. Anterior insula activity has also been highlighted as a common neural substrate across simulation of both social perception and mental imagery of disgust [6], overlapping with reported activation related to internal representations of pain such as empathy of pain in others [48] and expectation of pain [49]. Most recently, its cooperative role with the midcingulate in interoceptive representations has been extended more generally to human awareness [50]. This may explain why the anterior insula is not seen in a contrast of imagined pain > imagined warm, as this activity is not necessarily pain specific.

### Time-to-test Interval

Previous studies have suggested that a memory trace of a painful experience may exist but in a limited form such that sensory-re-experiencing is not available long after a painful event [16], [22], [23]. Derbyshire et al. however, found several subjects reported warming of the hand during a hypnotically induced imagined pain event [10]. These contradictory findings may simply be due to the time between the original and the recalled event shown to significantly influence retrospective judgement of past pain events [16], [51]. The short time interval in our study was chosen in the light of these findings and has clearly allowed accurate recall and simulation of the thermal pain experience with concomitant brain activity. Within the chosen time-to-test interval, a more complete trace of the physical pain stimulus is still available from which to create simulation of pain. Understanding how pain memory adapts over time might help explain the reported loss of sensory re-experiencing with longer intervals that can be adaptive in the context of pain [22].

### Conclusion

In our study we demonstrate that, without hypnosis, a recall task was sufficient to create a quantifiable sensory re-experiencing of pain that activated all of the classical pain structures except the contralateral posterior insula. Identifying one structure unique to processing nociceptive pain may aid in further characterising the differences between peripherally and centrally mediated pain experiences. We suggest that the imagined pain events were created by using a recent and retrievable memory trace, with structures in the working memory network enabling this retrieval. The concept of a pain trace may be helpful in better understanding the efficacy of therapies including hypnosis and mental imagery tasks [52], [53], possibly altering or replacing the patient's pain memory which is created, maintained and altered by pain experiences. It may theoretically therefore be useful to use to further explore a unifying concept of experience or trace-based simulation to better understand non-nociceptive components of the pain experience including anticipation, expectation and pain memory [54].
